# Differentiation of beta-like cells from human induced pluripotent stem cell-derived pancreatic progenitor organoids

**DOI:** 10.1016/j.xpro.2022.101656

**Published:** 2022-08-30

**Authors:** Sergio Pedraza-Arevalo, Ana-Maria Cujba, Mario Enrique Alvarez-Fallas, Rocio Sancho

**Affiliations:** 1 Center for Gene Therapy and Regenerative Medicine, Kings College London, London, SE1 9RT, UK; 2 Department of Internal Medicine III, University Hospital Carl Gustav Carus at the Technische Universität Dresden, Dresden, Germany

**Keywords:** Developmental biology, Metabolism, Stem cells, Cell differentiation, Organoids

## Abstract

Human induced pluripotent stem cells (hiPSCs) and organoids are important for modeling human development and disease *in vitro*. In this study, we describe a protocol to differentiate hiPSC toward pancreatic progenitor (PP) organoids and beta-like cells. We detail the expansion and seeding of hiPSC, PP differentiation, organoid expansion, and the differentiation of PP into beta cells. Upon differentiation, organoids contained beta, delta, and alpha cells.

For complete details on the use and execution of this protocol, please refer to Cujba et al. (2022).

## Highlights

• Step-by-step protocol for hiPSC differentiation to pancreatic progenitors
• Expansion of pancreatic progenitors in 3D organoids
• Differentiation of pancreas progenitor cell aggregates to beta-like cells
• Beta-like cell differentiation of pancreatic progenitor organoids in Matrigel domes

## Abstract (editor highlights)

Publisher’s note: Undertaking any experimental protocol requires adherence to local institutional guidelines for laboratory safety and ethics.

## Graphical abstract

**Figure:**
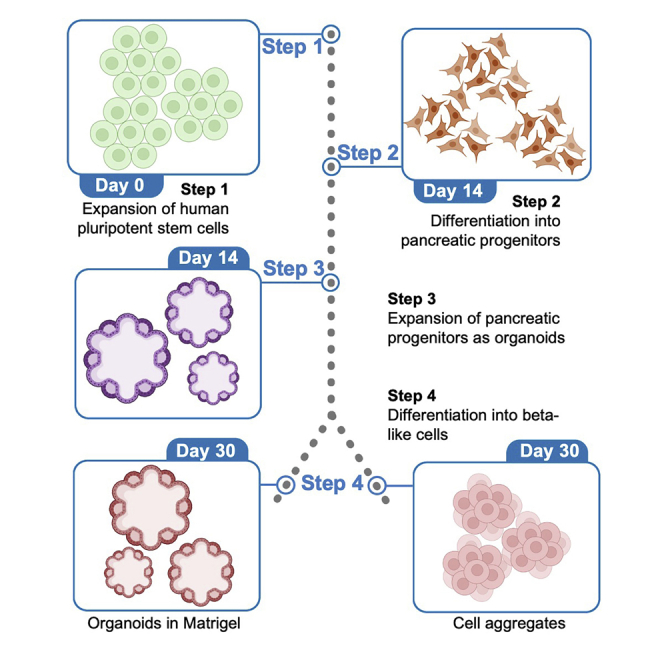

## Before you begin

**Figure 1:**
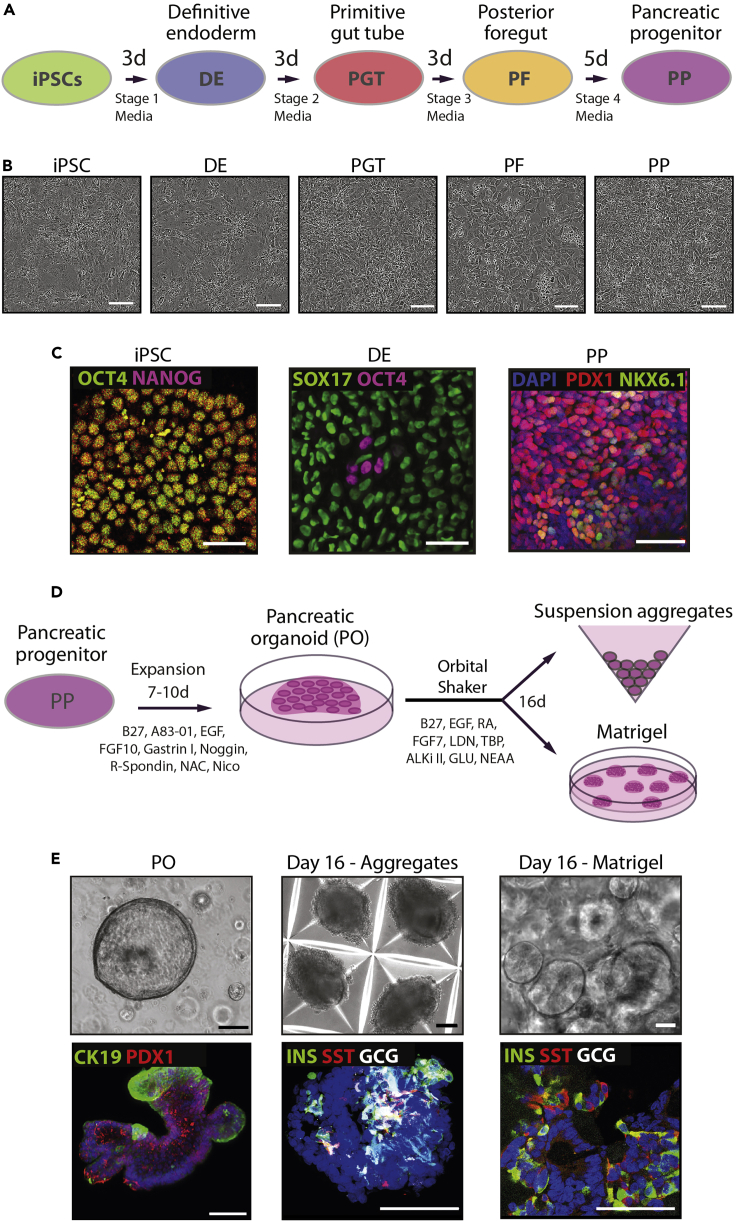
Differentiation summary (A) Schematic diagram of hiPSCs differentiated toward definitive endoderm (DE), primitive gut tube (PGT), posterior foregut (PF) and pancreatic progenitors (PP), with the respective brightfield (B) images. (C) Representative Immunofluorescence images at the iPSCs, DE and PP stages. (D) Schematic diagram of PPs expanded as pancreatic organoids (PO) and differentiated towards beta-like cells, either in suspension or Matrigel aggregates, with the respective brightfield and immunofluorescence pictures (E). Scale bars, 50 μm (B and C) and 100 μm (E). Adapted from Cujba et al. (2022).

The protocol below describes a method to differentiate beta-like cells from human induced pluripotent stem cells (hiPSCs) through an intermediary step of pancreas progenitor expansion as 3D organoids (Figure 1). This protocol is important to overcome the low efficiency and high complexity that common beta cell differentiation protocols have. The protocol presented here allows the researcher to improve the beta like cell yield using 3D-based culture conditions that promote the expansion of cells at an intermediate progenitor stage. Additionally, differentiating the cells in 3D Matrigel domes facilitate scaling up the experiment to increase the amounts of beta-like cells obtained. This differentiation recapitulates the key pancreatic developmental stages including definitive endoderm (DE), primitive gut tube (PGT), posterior foregut (PF), pancreatic progenitor (PP) and includes the intermediary pancreatic organoid (PO) expansion and final islet-like differentiation. The POs generated by this protocol can be frozen and thawed successfully (up to passage 15) (Cujba et al., 2022) and can be used for long term-experiments, saving cost and time. All the steps underlined bellow are carried out in sterile and aseptic conditions using a Class II safety laminar flow cabinet. Regular mycoplasma tests were carried out on all cell cultures to ensure no contamination took place during the culturing. The protocol bellow contains 4 main steps: 1) expansion and seeding of hiPSC for PP differentiation, 2) PP differentiation, 3) pancreatic organoid expansion, and 4) PP differentiation into beta-like cells. We start the hiPSC culture with one 6-well plate. Upon expansion, this yields approximately 9 million cells that can be used to differentiate a full 12-well plate and four 96 well plates (with 12 wells in each) for the validation of the four different stages of development throughout the protocol by immunofluorescence. Each user will need to scale up the cell numbers and plates to fulfill their needs.

### Preparation of vitronectin-coated plates for hiPSC expansion

**Timing: 1 h**

1. Pre-thaw the necessary amount vitronectin on ice for plate coating.
2. Mix 40 μL vitronectin with 1 mL of D-PBS to obtain a final concentration of 10 μg/mL.
3. Coat one well of a 6-well plate with vitronectin mix (1.04 mL).
4. Allow vitronectin coating to settle for 1 h at 20°C–25°C (expressed as RT over the manuscript).
5. Aspirate the mix.
6. Use the plate immediately or store up to a week at 2°C–8°C wrapped in parafilm to avoid it drying out.

### Preparation of medium for hiPSC expansion

**Timing: 1 h**

7. Prepare the amount of necessary Essential 8 Basal Medium (E8) culturing medium with supplement in advance, depending on the number of wells or cells needed.
  a. Thaw one vial of E8 supplement at RT for at least 30 min–1 h.
  b. Allow E8 medium to warm up at RT for at least 30 min–1 h.
  c. Mix E8 medium with the E8 supplement (50×) by adding 2 mL of supplement to 98 mL of E8 medium. The supplemented medium can be stored for 1 week at 2°C–8°C.
8. Each well of a 6-well plate requires 2 mL of medium that should be changed daily.

### Preparation of medium for hiPSC splitting

**Timing: 10 min**

9. Dilute 5 μL of sterile 0.5 M EDTA in 50 mL of D-PBS, to obtain the working concentration of 0.5 mM EDTA. This buffer can be stored at 2°C–8°C for 1 year.
10. This solution will detach the cells as colonies, and it is stable at RT for at least 4 weeks.

### Preparation and plating of iPSCs for differentiation

**Timing: 1 h**

11. Pre-thaw on ice the necessary amount of hESC-qualified Matrigel for the plate coating mix (0.12 mg/mL of Matrigel in cold DMEM medium, which usually is 1:80 dilution, but it depends on the Matrigel batch).
12. Pre-thaw Accutase for cell detachment at RT for 30 min.
13. Add 1 mL of coating mix to each well of a 6-well plate. Scale down if necessary.
14. Allow the Matrigel coating to settle for at least 1 h at RT.

**CRITICAL:** Matrigel must not exceed the temperature of 4°C for an extended amount of time to avoid irreversible solidification. Keep on ice until ready for intended application.

**CRITICAL:** Prepare complete mTeSR1 (medium plus supplement) and add Y-27632 to a final concentration of 10 μM.

### Preparation and plating of PP for expansion as organoids

**Timing: 6 h**

15. Pre-warm at 37°C the required number of 24-well plates to allow faster Matrigel solidification when plating progenitor organoids.
16. Pre-thaw stock solutions necessary to prepare complete medium.
17. Thaw the required amount of growth factor reduced Matrigel on ice,
18. Each well of a 24-well plate requires 25 μL of Matrigel.

**CRITICAL:** Matrigel must not exceed the temperature of 4°C for an extended time to avoid irreversible solidification. Keep on ice until ready for intended application.

## Key resources table

**Table:** 

REAGENT or RESOURCE	SOURCE	IDENTIFIER
**Antibodies**		

Goat anti-SOX17 (1:200)	R&D	Cat#AF1924; RRID: AB_355060
Mouse anti-OCT4 (1:300)	Santa Cruz	Cat#sc-5279; RRID: AB_628051
Rabbit anti-NANOG (1:300)	Cell Signaling Technology	Cat#4903S; RRID: AB_10559205
Rabbit anti-PDX1 (1:300)	Cell Signaling Technology	Cat#5679; RRID: AB_10706174
Guinea pig anti-PDX1 (1:300)	Abcam	Cat#ab47308; RRID: AB_777178
Mouse anti-NKX6.1 (1:400)	DSHB	Cat#F55A12; RRID: AB_532379
Goat anti-SOX9 (1:300)	R&D	Cat#AF3075; RRID: AB_2194160
Guinea pig anti-insulin (1:300)	Dako	Cat#A0564; RRID: AB_10013624
Rabbit anti-somatostatin (1:300)	Dako	Cat#A0566; RRID: AB_2688022
Mouse anti-glucagon (1:300)	Abcam	Cat#ab10988; RRID: AB_297642
Rat anti-CK19 (1:300)	DSHB	Cat#TROMA IIIc 10ea; RRID: AB_2133570
Donkey anti-mouse Alexa 647 (1:300)	Invitrogen	Cat#A31571; RRID: AB_162542
Donkey anti-rabbit Alexa 488 (1:300)	Invitrogen	Cat#A21206; RRID: AB_2535792
Donkey anti-goat Alexa 647 (1:300)	Invitrogen	Cat#A21432; RRID: AB_2535853
Donkey anti-mouse Alexa 488 (1:300)	Invitrogen	Cat#A21202; RRID: AB_141607
Goat anti-guinea pig Alexa 488 (1:300)	Invitrogen	Cat#A11073; RRID: AB_2534117
Donkey anti-rat Alexa 647 (1:300)	Invitrogen	Cat#A21472; RRID: AB_1500700

**Chemicals, peptides, and recombinant proteins**		

Ethylenediaminetetraacetic acid (EDTA)	Fisher Bioreagents	BP2482-500
Accutase	BioLegend	423201
Matrigel Growth Factor Reduced (GFR) Basement Membrane Matrix	Corning	354230
Vitronectin	STEMCELL Technologies	7180
HEPES	Thermo Fisher Scientific	15630080
Glutamax supplement	Thermo Fisher Scientific	3505006
Penicilin/Streptomycin	Thermo Fisher Scientific	15140122
A83-01	Tocris	2939
Human FGF-10	PeproTech	100-26
GastrinI	Sigma	SCP0151
Mouse EGF	PeproTech	AF10015
N-acetylcysteine	Sigma-Aldrich	A9165
Nicotinamide	Sigma-Aldrich	N0636-100G
B-27 supplement	Thermo Fisher Scientifi	17504044
Noggin conditioned medium	This study	N/A
R-spondin conditioned medium	This study	N/A
Y-27631	Adooq Bioscience	A11001
TrypLE	Thermo Fisher Scientific	12604021
Retinoic acid	Sigma	R2625-50MG
LDN-193189	Stemgent	04-0074
TBP	Millipore	565740-1MG
ALKi II	Axxora	ALX-270-445-M001
Non-Essential Amino Acids	Gibco	11140035
FGF7	R&D Systems	251-KG-010

**Critical commercial assays**		

STEMdiff™ Pancreatic Progenitor Kit	STEMCELL Technologies	05120

**Experimental models: Cell lines**		

Wild-type Human iPSC line, from skin tissue of a female donor	HipSci	HPSI0714i-kute_4

**Other**		

Essential 8 Basal Medium	Gibco	A15169-01
Advanced DMEM/F-12	Gibco	12634-010
Cell recovery solution	Corning	354253
Aggrewell™ 400 plates	STEMCELL Technologies	34411
DMEM 2.8 mM glucose	Gibco	21885025
DMEM 25 mM glucose	Gibco	31966021
DAPI	Invitrogen	D1306

## Materials and equipment

**Table:** Organoid expansion medium

Reagent	Final concentration	Amount
A83-01 (0.5 mM)	0.5 nM	50 μL
B-27 (50×)	1×	1 mL
EGF (50 μg/mL)	50 ng/mL	50 μL
FGF10 (100 μg/mL)	50 ng/mL	50 μL
Gastrin 10 μM	10 mM	50 μL
Noggin (10×)	1×	5 mL
N-Acetylcysteine (500 mM)	1.25 mM	125 μL
Nicotinamide (1 M)	10 mM	500 μL
Penicillin-Streptomycin (100×)	1×	500 μL
R-Spondin (10×)	1×	5 mL
Glutamax (100×)	1×	500 μL
HEPES (100×)	1×	500 μL
Advanced DMEM/F-12	N/A	36.675 mL
**Total**	**N/A**	**50 mL**

**Table:** Medium for stage 1 of pancreatic progenitors differentiation to beta cells

Reagent	Final concentration	Amount
B-27 (50×)	0.5×	100 μL
EGF (50 μg/mL)	50 ng/mL	10 μL
Retinoic acid (10 mM)	1 μM	1 μL
DMEM 25 mM Glucose	N/A	9.889 mL
**Total**	**N/A**	**10 mL**

***Note:*** This medium must be prepared with double concentration of the reagents to feed the aggregates.

**Table:** Medium for stage 2 of pancreatic progenitors differentiation to beta cells

Reagent	Final concentration	Amount
B-27 (50×)	0.5×	100 μL
EGF (50 μg/mL)	50 ng/mL	10 μL
FGF7 (100 mg/mL)	50 ng/mL	5 μL
DMEM 25 mM Glucose	N/A	9.885 mL
**Total**	**N/A**	**10 mL**

***Note:*** This medium must be prepared with double concentration of the reagents to feed the aggregates.

**Table:** Medium for stage 3 of pancreatic progenitors differentiation to beta cells

Reagent	Final concentration	Amount
B-27 (50×)	0.5×	100 μL
LDN-193189 (5 mM)	500 nM	1 μL
TBP (300 μM)	30 nM	1 μL
ALKi II (10 mM)	1 μM	1 μL
FGF7 (100 mg/mL)	25 ng/mL	2.5 μL
DMEM 25 mM Glucose	N/A	9.8945 mL
**Total**	**N/A**	**10 mL**

***Note:*** This medium must be prepared with double concentration of the reagents to feed the aggregates.

**Table:** Medium for stage 4 of pancreatic progenitors differentiation to beta cells

Reagent	Final concentration	Amount
Glutamax (100×)	1×	100 μL
Non-essential amino acids (100×)	1×	100 μL
DMEM 2.8 mM Glucose	N/A	9.8 mL
**Total**	**N/A**	**10 mL**

***Note:*** This medium must be prepared with double concentration of the reagents to feed the aggregates.

## Step-by-step method details

### Step 1: Expansion and plating of human iPSCs

**Timing: 5–10 days**

This section describes the procedure for thawing and expanding human iPSCs (hiPSCs), which will then be used for pancreatic progenitor (PP) differentiation. Human iPSCs are cultured under feeder-free conditions and should be passaged once the culture reaches approximately 70% confluency.

1. Prepare 6-well plates coated with vitronectin prior to thawing an iPSCs frozen vial as described below. A 6-well plate is sufficient for one differentiation, with approximately 80% confluency and 1–1.5 million cells obtained per well at the end of the differentiation.
  a. Thaw a vial of 2 mL vitronectin at RT. Once thawed, make aliquots for 3 wells (120 μL) and 6 wells (240 μL) of a 6-well plate. Aliquots can be kept at −20°C for long-term storage.
  b. Dilute vitronectin in D-PBS by adding 40 μL of vitronectin per 1 mL of D-PBS, to a final concentration of 10 μg/mL. Scale it up accordingly for the necessary wells needed in the 6-well plate. This should be prepared fresh and used at the same day.
  c. Add 1 mL diluted vitronectin to each well. Swirl the volume across the well to cover the entire surface and incubate the plate at room-temperature in the laminar flow cabinet.
  d. Aspirate the vitronectin before seeding the cells.

***Note:*** Do not let the plate dry before seeding the cells, only remove the coating just before adding the cell suspension.

2. Thaw a cryovial of human iPSC by gently swirling it in a pre-warmed 37°C water bath until there is only a small blob of ice remaining in the vial.
3. Dry and spray the cryovial with 70% ethanol.
4. In a laminar flow cabinet, transfer the cell suspension gently with a 1 mL pipette to a 15 mL conical tube containing 5 mL of E8 culture medium supplemented with 10 mM Y-27632.
5. Add 1 mL of E8 + Y-27632 solution dropwise to the cryovial with a 5 mL stripette, then gently collect and transfer the entire cell suspension to a 15 mL Falcon tube. This should be prepared fresh and used at the same day.
6. Centrifuge the cells at 300 g for 3 min at RT.
7. During this time, aspirate vitronectin from the 6-well coated culture plate.
8. After centrifugation remove the supernatant and resuspend the cell pellet in 6–12 mL E8 + Y-27632 and transfer 2 mL into each of the vitronectin-coated wells.
9. Swirl the plate gently to ensure even distribution of cells across the wells and place it in a tissue culture incubator set at 37°C and 5% CO_2_.
10. Check cell attachment under a phase contrast microscope after 24 h.
11. If attachment is good, replace the medium with 2 mL E8 without Y-27632. If there are more cells floating than attached, top up with 1 mL freshly made E8 culture medium containing 10 mM Y-27632. This should be prepared fresh and used at the same day.

**Figure 2:**
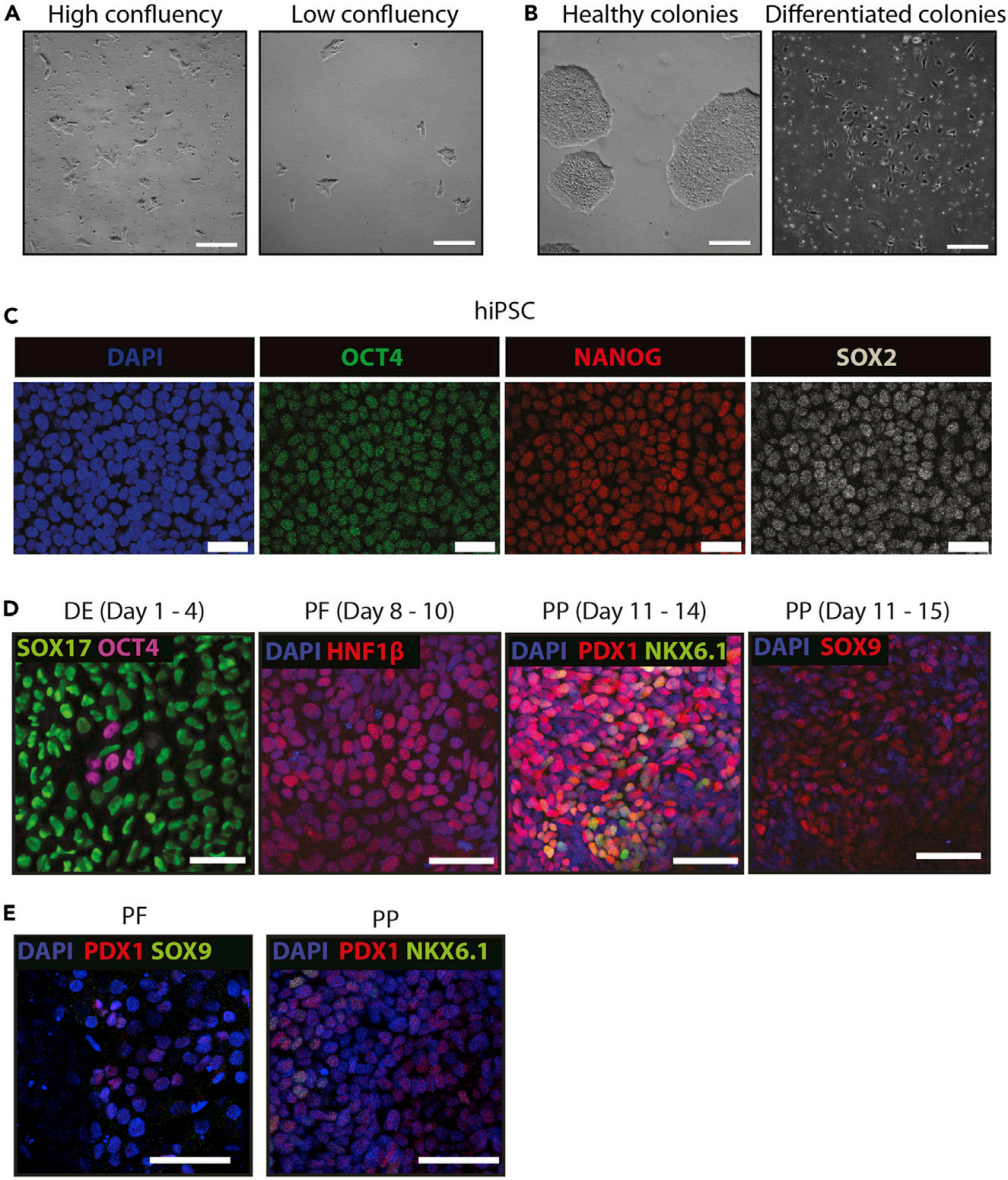
Characterization of pancreatic progenitors (PP) (A) Brightfield images of a hiPSC culture at high (left) and low (right) confluency, one day after splitting. (B) Brightfield images of a healthy hiPSC culture with defined edges (left) and a differentiated unhealthy hiPSC culture with stromal-like morphologies and heterogenous cells (right). (C) Immunofluorescence of hiPSC at the beginning of the differentiation, validated for expression of the pluripotency markers OCT4, NANOG, and SOX2. (D) Immunofluorescence for DE markers SOX17 and pluripotent marker OCT4 at the DE stage, HNF1B at the PF stage, and PDX1, NKX6.1, and SOX9 at the PP stage. (E) Example of a suboptimal differentiation characterized by few and low expressing PDX1, NKX6.1 and SOX9 positive cells at the PP stage. Scale bars, 50 μm (A, B, D and E) and 30 μm (C). Adapted from Cujba et al. (2022).

***Note:*** Example of healthy hiPSCs and differentiated unhealthy colonies are shown in Figure 2B. If hiPSC colonies look differentiated, the best colonies can be selected for further expansion by manual picking with a pipette, splitting and replating, or use of ReLeSR. However, it is recommended to rethaw new cells if colonies look differentiated and unhealthy.

***Note:*** We recommend splitting the cells from a cryovial frozen from one well (equivalent to 1 million cells) into 3 wells with a higher density (Figure 2A, left) and 3 wells with a lower density (Figure 2A, right), to monitor how different cells recover upon thawing.

12. Medium change should be done every day for best results with E8 cell culture medium. Once per passage, cells may be double-fed (4 mL) for two days. The cells will be ready to be passaged in about 3–5 days, depending on the growth rate and recovery of the cells.

13. Once the colonies reach about 70% confluency, proceed to cell passaging by coating a full 6-well plate with Vitronectin. Cells may also require passaging if levels of differentiation start to exceed that of iPSC or colonies start to look overgrown or unhealthy. One 6-well plate could be directly used for the human iPSC differentiation.

***Note:*** Count the number of total cells needed for the different conditions (500,000 cells per well of a 12-well plate or 42,000 cells per well of a 96-well plate) prior to setting up the necessary plates through detailed experimental design.

14. Established iPSCs cultures can be split 1:4 to 1:6 (i.e., splitting colonies from one well to four or six wells, respectively).
15. Wash cells with 2 mL of D-PBS.
16. If a high proportion of differentiated cells is observed, scrap and remove huge clusters of differentiated cells under the laminar flow hood microscope using sterile tips (only if necessary).
17. Add 1 mL of 0.5 mM EDTA solution (tissue culture grade) and swirl the plate gently to cover all the surface.
18. Incubate the plate at RT for 4–8 min or in the incubator at 37°C for 3–5 min. Observe them under a phase contrast microscopy until colonies display bright halos around the edges and small holes start to appear throughout the colonies indicating detachment.
19. Aspirate the EDTA solution.
20. Use a 1 mL pipette and add 2 mL E8 medium on top of the cells to detach them with mechanical force.
21. Transfer the colonies to a 15 mL tube.

***Note:*** There is a tendency for the cells to differentiate more at the edges of the well, hence try to detach the healthy ones from the middle of the well. There is no need to passage all the cells, as the first passage after thawing usually does not expands the healthiest of the colonies.

22. Incubate the 15 mL tube for 3 min at RT inside the laminar flow cabinet.

***Note:*** The undifferentiated clusters will sink first at the bottom of the tube while the most differentiated cells will stay for longer at the top of the tube due to gravity, ensuring a physical separation of the differentiated versus non-differentiated cells.

23. Carefully, aspirate the single cells at the top of the tube and leave the clusters that sunk down.
24. Add 1 mL of E8 medium on top of the cells and resuspend once or twice slowly to make smaller clusters.

**CRITICAL:** Avoid generating a single-cell suspension by resuspending too harshly or too many times. The gentler the pipetting is, the most likely the small clusters will recover well upon passaging. Single cells obtained by harsh culturing conditions will likely result in differentiated colonies and poor cell survival.

25. Add around 3–6 drops of the cell suspension/per well onto a new plate coated with vitronectin. Adjust cell density as necessary, but usually a lower density allows for growth of healthier colonies.
26. Swirl plate gently and incubate at 37°C and 5% CO_2_.
27. Change the culture medium daily.
28. The newly passaged 6-well plate should reach 80%–90% confluency in 5–6 days and can be used to start the iPSC differentiation.

**CRITICAL:** Ensure there are no differentiated colonies in these expanding hiPSC cultures. These colonies show an elongated shape and heterogenous morphologies (Figure 2B, right) compared to the homogenous healthy hiPSC colonies (Figure 2B, left). If the differentiated cells only represent a small percentage of the cell culture (<10%), remove them under sterile conditions by scratching with a sterile pipette tip or use of ReLeSR to improve hiPSC passaging. It is also possible to manually pick healthy cell colonies scraping them with a pipette and passing then to a new well. If cell cultures exhibit a big percentage of differentiated cells (>10%), start a new culture.

***Note:*** The section below is written and adapted from the original commercial publication (STEMdiff™ Pancreatic Progenitor Kit, STEMCELL Technologies, https://www.stemcell.com/products/stemdiff-pancreatic-progenitor-kit.html) and contains small changes optimized to work with our cell lines.

29. Prepare one 12-well plate coated with human-ESC grade Matrigel matrix for flow cytometry, RT-qPCR analysis, and organoid expansion. In addition, prepare 4 black Greiner 96-well plates for imaging for the 4 differentiation stages: definitive endoderm (DE), primitive gut tube (PGT), posterior foregut (PF), and pancreatic progenitor (PP).
  a. Thaw one vial of hESC-grade Matrigel at 2°C–8°C for 16 h or on ice for at least 4 h. Once thawed, make smaller aliquots using refrigerated pipette tips and store them at −20°C long-term.
    ***Note:*** Matrigel is very sensitive to room temperatures, resulting in instant gelling. Always use ice and pre-chilled freezer tube rack for aliquoting and coating plates.
  b. Once Matrigel is thawed, mix it with cold DMEM/F-12 medium in the recommended dilution. The final concentration of Matrigel should be 0.12 mg/mL, which approximately will be 12 μL of Matrigel per mL of medium. However, the dilution must be calculated for each lot based on the protein concentration. This mix can be stored at 2°C–8°C for 1 week.
  c. For coating, use 500 μL/well of a 12-well plate for coating and 100 μL/well for a 96-well plate.
  d. Incubate the plates for at least 1 h at RT or in the incubator under sterile conditions.
30. Warm at RT mTeSR™1 medium, DMEM/F-12 and Accutase for hiPSC cell seeding.
31. Add Y-27632 to mTeSR™1 to a final concentration of 10 μM to make single-cell passaging medium. This should be prepared fresh and used at the same day.
32. Wash the expanded hiPSCs with 1 mL of D-PBS.
33. Aspirate D-PBS and add 1 mL of Accutase.
34. Incubate at 37°C for 8–10 min. Depending on the cell line, this step might take only 3–5 min; optimize this for your cell line.
35. Collect the cells by pipetting up and down 1–3 times using a pipettor with a 1 mL tip. Make sure all remaining cell aggregates are broken up into single cells.
36. Immediately transfer cells to a tube with an equal volume of DMEM/F-12. Wash the well with 1 mL of DMEM/F-12 to take any remaining cells and transfer to the tube. Centrifuge the tube at 300 g for 5 min at RT.
37. Resuspend cells in 1 mL of single cell passaging medium and count the cells with a hemocytometer.
38. Add cells at a density of 5 × 10^5^ cells/well (for 12-well plates) and 0.42 × 10^5^ cells/well (for 96-well plates) to coated plates.
39. Incubate at 37°C and 5% CO_2_ for 24 h in the tissue culture incubator.

***Note:*** After 24 h, considerable cell death will be observed in the wells following attachment, this is normal. Wash the cells with HBSS before adding the induction medium to avoid carryover of dead cells.

### Step 2: Differentiation of human iPSCs to pancreatic progenitors

**Timing: 15 days**

This section describes the differentiation of expanded human iPSCs to pancreatic progenitors in 2D using the STEMdiff™ Pancreatic Progenitor Kit according to manufacturer’s instructions with small adjustments. The differentiation can be assessed by the formation of PDX1+/NKX6.1+ pancreatic progenitors (Figure 2).

***Note:*** The section below is written and adapted from the original commercial publication (STEMdiff™ Pancreatic Progenitor Kit, STEMCELL Technologies, https://www.stemcell.com/products/stemdiff-pancreatic-progenitor-kit.html) and contains small changes optimized to work with our cell lines.

***Note:*** 6 different media are required for the 4 stages of the differentiation protocol, including Definitive Endoderm (DE, Stage 1), Primitive Gut Tube (PGT, Stage 2), Posterior Foregut (PF, Stage 3), Pancreatic Progenitor (PP, Stage 4). Prepare each medium as described below. Medium 1A and Medium 1B may both be prepared on Day 1 of Stage 1; Medium 2A and Medium 2B may both be prepared on Day 1 of Stage 2. Store prepared media at 2°C–8°C until warmed at 37°C for use. After preparing the required volumes of media, remaining supplements should aliquoted and stored at −20°C to avoid unnecessary freezing-thawing cycles. Do not exceed the expiration date of the supplements. Supplements must be used immediately after thawing. Do not re-freeze.

#### *Stage 1*: Differentiation to definitive endoderm

**Timing: 3 days**

40. Make the necessary volume of Medium 1A (Endoderm Basal Medium + Supplement MR + Supplement CJ) for Day 1 of Stage 1 (800 μL/12-well plate and 200 μL/96-well plate).
  a. Thaw entire bottle of Stage 1 Basal Medium at RT or at 2°C–8°C for 16 h.
  b. Mix thoroughly.
    ***Note:*** If not used directly, store at 2°C–8°C for up to 2 months or aliquot and store at −20°C. Do not exceed the expiration date of the medium. Aliquots must be use immediately after thawing or stored at 2°C–8°C for up to 2 weeks. Do not re-freeze.
  c. For 1 mL medium, on Day 1 of Stage 1, warm at 37°C 1 mL of Endoderm Basal Medium.
  d. Thaw Supplements MR and CJ on ice.
  e. Add 10 μL of each supplement to 0.99 mL of Endoderm Basal Medium.
  f. Mix thoroughly and use immediately.
41. Aspirate medium from wells and replace with (800 μL/12-well plate and 200 μL/96-well plate of warm (37°C) Medium 1A per well.
42. Incubate plate at 37°C and 5% CO_2_ for 24 h in the tissue culture incubator.
43. Prepare the volume of Medium 1B (Endoderm Basal Medium + Supplement CJ) required for Day 2 and Day 3 of Stage 1.
  a. For 2 mL medium, add 20 μL of cold (2°C–8°C) Supplement CJ to 1.98 mL of cold (2°C–8°C) Endoderm Basal Medium.
  b. Mix thoroughly and store the rest of the medium at 2°C–8°C until the day of intended use.
44. On day 2 of Stage 1, change medium to pre-made and pre-warmed (37°C) Medium 1B.
45. Incubate plate at 37°C and 5% CO_2_ for 24 h in the tissue culture incubator.
46. On day 3 of Stage 1, change medium to pre-made and pre-warmed (37°C) Medium 1B.
47. Incubate plate at 37°C and 5% CO_2_ for 24 h in the tissue culture incubator.

***Note:*** Differing from the original protocol, we have increased stage 1 from two days to three days, to allow better differentiation and recovery of our cell lines. It is normal to observe cell death in the first stage. To avoid carryover of dead cells, they can be removed by quick washes with pre-warmed HBSS between changing medium daily for this stage.

#### *Stage 2*: Differentiation to primitive gut tube

**Timing: 3 days**

48. Make the necessary volume of Medium 2A (Stage 2–4 Basal Medium + Supplement 2A + Supplement 2B) for Day 1 of Stage 2 (800 μL/12-well plate and 200 μL/96-well plate).
  a. For 1 mL medium, on Day 1 of Stage 2, warm (37°C) 1 mL of Stage 2–4 Basal Medium.
  b. Thaw Supplement 2A and Supplement 2B on ice.
  c. Add 10 μL of Supplement 2A and 10 μL of Supplement 2B to 0.99 mL of Stage 2–4 Basal Medium.
49. Mix well and use immediately.
50. Aspirate medium from wells and replace with 800 μL/12-well plate and 200 μL/96-well plate of warm (37°C) Medium 2A per well.
51. Incubate plate at 37°C and 5% CO_2_ for 24 h in the tissue culture incubator.
52. Prepare the volume of Medium 2B (Stage 2–4 Basal Medium + Supplement 2B) necessary for Days 2 and 3 of Stage 2.
  a. For 2 mL Medium 2B, add 20 μL of cold (2°C–8°C) Supplement 2B to 1.98 mL of cold (2°C–8°C) Stage 2–4 Basal Medium.
  b. Mix thoroughly and store the rest of the medium at 2°C–8°C until the day of intended use.
53. On day 2 of Stage 2, change medium to pre-made and pre-warmed (37°C) Medium 2B.
54. Incubate plate at 37°C and 5% CO_2_ for 24 h in the tissue culture incubator.
55. On day 3 of Stage 2, change medium to pre-made and pre-warmed (37°C) Medium 2B.
56. Incubate plate at 37°C and 5% CO_2_ for 24 h in the tissue culture incubator.

#### *Stage 3*: Differentiation to posterior foregut

**Timing: 3 days**

57. Prepare the volume of Medium 3 (Stage 2–4 Basal Medium + Supplement 3) required for Days 1, 2, and 3 of Stage 3.
  a. For 3 mL of Medium 3, on Day 1 of Stage 3, thaw Supplement 3 on ice.
  b. Add 30 μL of Supplement 3 to 2.97 mL of cold (2°C–8°C) Stage 2–4 Basal Medium.
  c. Mix well and use immediately.
58. Aspirate medium from wells and replace with 800 μL/12-well plate and 200 μL/96-well plate of warm (37°C) Medium 3 per well.
59. Incubate at 37°C and 5% CO_2_ for 24 h in the tissue culture incubator.
60. Mix well and store the rest of the medium at 2°C–8°C until the day of intended use.
61. On day 2 of Stage 3, change medium to pre-made and pre-warmed (37°C) Medium 3.
62. Incubate plate at 37°C and 5% CO_2_ for 24 h in the tissue culture incubator.
63. On day 3 of Stage 3, change medium to pre-made and pre-warmed (37°C) Medium 3.
64. Incubate plate at 37°C and 5% CO_2_ for 24 h in the tissue culture incubator.

#### *Stage 4*: Differentiation to pancreatic progenitors

**Timing: 3 days**

65. Prepare the required volume of Medium 4 (Stage 2–4 Basal Medium + Supplement 4) required for Days 1, 2, and 3 of Stage 4.
  a. For 3 mL Medium 4, on Day 1 of Stage 4, thaw Supplement 4 on ice.
  b. Add 30 μL of Supplement 4 to 2.97 mL of cold (2°C–8°C) Stage 2–4 Basal Medium.
  c. Mix well and use immediately.
66. Aspirate medium from wells and replace with 800 μL/12-well plate and 200 μL/96-well plate of warm (37°C) Medium 4 per well.
67. Leave the plate at 37°C and 5% CO_2_ for 24 h in the tissue culture incubator.
68. Store the rest of the medium at 2°C–8°C until intended use.
69. On day 2 of Stage 4, change medium to pre-made and pre-warmed (37°C) Medium 4.
70. Incubate plate at 37°C and 5% CO_2_ for 24 h in the tissue culture incubator.
71. On day 3 of Stage 4, change medium to pre-made and pre-warmed (37°C) Medium 4.
72. Incubate plate at 37°C and 5% CO_2_ for 24 h in the tissue culture incubator.
73. Aliquot the volume required of Supplement 4 for Day 4 of Stage 4 and store the rest at −20°C.
74. On Day 4, make the required volume of Medium 4 (Stage 2–4 Basal Medium + Supplement 4) for Days 4 and 5 of Stage 4.
75. For 2 mL of Medium 4, on Day 4 of Stage 4, thaw the aliquoted Supplement 4 on ice.
76. Add 20 μL of cold (2°C–8°C) Supplement 4 to 1.98 mL of cold (2°C–8°C) Stage 2–4 Basal Medium.
77. Mix well and use immediately.
78. Aspirate medium from wells and replace with 800 μL/12-well plate and 200 μL/96-well plate of warm (37°C) Medium 4 per well.
79. Leave the plate at 37°C and 5% CO_2_ for 24 h in the tissue culture incubator.
80. Store the rest of the medium at 2°C–8°C until intended use.
81. On day 5 of Stage 4, change medium to pre-made and pre-warmed (37°C) Medium 4.
82. Incubate plate at 37°C and 5% CO_2_ for 24 h in the tissue culture incubator.
83. Next day is the end of the differentiation.

### Step 3: Establishing 3D organoid culture and expansion of progenitors

**Timing: 1 day, then passaging every 7–10 days**

This section describes the establishment and maintenance of 3D organoid culture using pancreatic progenitors from differentiated hiPSCs. Successful establishment is based on organoid formation, batch quality of the culture is evaluated by organoid morphology.

***Note:*** Volumes and procedure are intended for a single well of a 12-well plate.

#### *Part 1*: Establishing 3D organoid culture

84. Prepare organoid expansion medium.
  a. Advanced DMEM/F-12 with 1× hepes, 1× glutamax, 1× penicillin-streptomycin, 1× B-27, 0.5 nM A83-01, 50 ng/mL EGF, 100 ng/mL FGF10, 10 nM GastrinI, 1× Noggin, 1× R-Spondin, 1.25 mM N-Acetylcysteine and 10 mM Nicotinamide; it can be stored at 2°C–8°C for one week.
85. Wash the pancreatic progenitors using 2 mL D-PBS, make sure you are very gentle, as the progenitors can easily detach at this stage after 15 days in culture.
86. Detach cells using 500 μL of Accutase for 5–10 min at 37°C, check when they are detached under the microscope.
87. Neutralize with 5% FBS in D-PBS. This mix can be stored at 2°C–8°C for 1 month.
88. Transfer the cell suspension to a tube and centrifuge at 300 g for 5 min at RT.
89. Aspirate supernatant carefully.
90. Resuspend the cell pellet in 300 μL Matrigel and leave it on ice for 5 min to remove bubbles.
91. Add 25 μL of the Matrigel cell culture suspension to the center of a well of a 24-well plate.
92. Incubate plates for 30–45 min at 37°C to allow solidification.
93. Add 500 μL of expansion medium + 10 μM Y-27631 to each well for the first 2–3 days. This should be prepared fresh and used at the same day.
94. Change medium every 2–3 days excluding Y-27631.

#### *Part 2*: Passaging the organoids

***Note:*** Organoids are usually expanded for 7–10 days before splitting. The split ratio differs due to growth, but usually one well can be split 1:4 or 1:6.

95. Aspirate medium and wash twice in D-PBS.
96. Add 250 μL TrypLE per well and dislodge Matrigel domes with syringe plunger.
97. Collect domes in a 2 mL Eppendorf tube (4 domes per tube).
98. Incubate on a Thermomixer™ at 37°C for 5 min at a mixing frequency of 600 RPM (check dissociation of the Matrigel and vortex every 2 min).
99. Add the same volume of D-PBS than TrypLE (for four domes, add 1 mL D-PBS).
100. Centrifuge at 300 g for 5 min at RT.
101. Aspirate the supernatant.
102. Resuspend pellet well in D-PBS.
103. Centrifuge at 300 g for 5 min at RT.
104. Aspirate the supernatant.
105. Resuspend pellet in Matrigel gently to avoid air bubbles and place the tube on ice. As an example, for an Eppendorf containing 4 digested domes, 400 μL of Matrigel should be used to resuspend the pellet and to plate 16 wells in a new 24-well plate.
106. Plate 25 μL of the Matrigel/cells mix per well in a new 24-well plate.

***Note:*** Using a hot heat pad under the plate can help the Matrigel to from a proper dome in the center of the well.

107. Incubate for 30–45 min at 37°C for Matrigel to polymerize.
108. Add 600 μL of organoid expansion medium per well.
109. Expand as described before for 7–10 days, at 37°C and 5% CO_2_ in the tissue culture incubator, changing the medium every other day.

**CRITICAL:** Successful outcome of later differentiation into endocrine cells depends on the quality of the cells in culture. A small percentage of mesenchymal cells is expected but should be kept at minimum (5% of total cells max). Since they are single cells, size separation based on shorter TrypLE incubation and centrifugation, or selection based on E-Cadherin can be performed.

#### *Part 3*: Freezing the organoids

***Note:*** Try not to expand the organoids beyond passage 8–10, they can stop proliferating as efficiently and accumulate other cell types in culture. Preferably, freeze some vials at early passages.

110. Aspirate medium and wash twice in D-PBS.
111. Add 250 μL TrypLE per well and dislodge Matrigel domes with syringe plunger.
112. Collect domes in a 2 mL Eppendorf tube (4 domes per tube).
113. Incubate on a Thermomixer™ at 37°C for 5 min at a mixing frequency of 600 RPM (check dissociation of the Matrigel and vortex every 2 min).
114. Add the same volume of D-PBS than TrypLE.
115. Centrifuge at 300 g for 5 min at RT.
116. Aspirate the supernatant.
117. Resuspend pellet well in D-PBS.
118. Centrifuge at 300 g for 5 min at RT.
119. Aspirate the supernatant.
120. Re-suspend in the required volume of organoid expansion medium (900 μL per vial to be frozen).
121. Dispense 900 μL of cell suspension into each cryovial and seal tightly.
122. Add 100 μL of DMSO and mix gently.
123. Immediately, place the cryovials into a pre-chilled cell freezing container (2°C–8°C) then transfer the container to a −80°C freezer. Leave the cells at −80°C for 16–36 h. Once frozen, transfer the cell to an Ultra-Low Temperature storage vessel (liquid nitrogen or −150°C freezer).

### Step 4A: Differentiation of pancreatic progenitors into beta cells as aggregates

**Timing: 17 days**

This section describes the differentiation of expanded pancreatic progenitors into beta cells using 24-well Aggrewell™ 400 plates. This protocol is an adaptation from (Russ et al., 2015; Trott et al., 2017), optimized for our cell lines.

124. Prepare and warm expansion medium.
125. Pre-treat the necessary wells of a 24 wells Aggrewell™ 400 plate with 500 μL of Anti-Adherence Rinsing Solution per well.
126. Centrifuge the plate at 300 g for 5 min and make sure there are no bubbles in the microwells.

***Note:*** Wells of Aggrewell™ 400 plates contain microwells of 400 μm that are useful to generate cell aggregates.

***Note:*** Anti-Adherence Rinsing Solution is required to prevent cell adhesion to the plate, increasing efficiency in cell aggregates production and spheroid formation.

127. Aspirate Anti-Adherence Rinsing Solution.
128. Rinse wells with 2 mL of expansion medium.
129. Add 1 mL of expansion medium with 10.5 μM Y-27631. This should be prepared fresh and used at the same day.
130. Split the organoids into single cell suspension.
  a. Aspirate the medium from the wells containing the domes of Matrigel with the organoids.
  b. Wash the wells with 500 μL of D-PBS.
  c. Add 250 μL of TrypLE per well, pipette up and down 3–5 times until the dome of Matrigel is disintegrated and collect the cells in a 1.5 mL Eppendorf tube.
  d. Rock the tube at 37°C for 7 min.
  e. Add 250 μL of D-PBS and pipette up and down to disaggregate the organoids.
  f. Spin the cells down for 5 min at 300 g at RT.
  g. Resuspend the cells in 1 mL of expansion medium with 10.5 μM Y-27631.
  h. Count the cells using a hemocytometer.
131. Seed 375,000 cells per well in the Aggrewell™ 400 plate.
132. Add expansion medium with 10.5 μM Y-27631 to achieve 2 mL per well.
133. Pipette up and down 5–8 times to ensure an appropriate distribution of the cells in the microwells, being careful not to introduce any bubbles.
134. Centrifuge the plate at 300 g for 3 min at RT to concentrate the cells in the microwells.

***Note:*** Use a standard plate with the same volume of water to balance the centrifuge.

135. Observe the cells under the microscope after every change of medium to verify an even distribution of the cells.
136. Incubate the plate for 24 h at 37°C and 5% CO_2_ before starting the differentiation.

#### *Stage 1:* Differentiation to pancreatic progenitors 2

**Timing: 2 days**

137. Prepare the volume of Stage 1 medium required for day 1 and 2: DMEM 25 mM Glucose with 0.5× B-27, 50 ng/mL EGF, 1 μM retinoic acid. 1 mL of medium per well and day with double concentration of reagents.

***Note:*** Medium change is done by removing only 1 mL and adding another mL of fresh medium, so medium must be prepared with double concentration of reagents.

***Note:*** Medium can be prepared in advance and stored for 1 day at 4°C.

138. Perform a 50% of medium change by gently removing 1 mL of medium from the well. Add 1 mL of fresh double concentrated medium by slowly pipetting in the wall of the well.
139. Incubate the plate at 37°C and 5% CO_2_ in an orbital shaker at 100 RPM inside the incubator for 24 h.

***Note:*** Medium change is performed daily, and cells are incubated in an orbital shaker at 100 RPM inside the incubator until the end of the differentiation. Warm medium before every change.

140. On day 2, change medium to pre-made and pre-warmed medium.
141. Incubate at 37°C and 5% CO_2_ in an orbital shaker at 100 RPM inside the incubator for 24 h.

#### *Stage 2:* Differentiation to endocrine pancreas

**Timing: 2 days**

142. Prepare the volume of Stage 2 medium required for day 1 and 2: DMEM 25 mM Glucose with 0.5× B-27, 50 ng/mL EGF, 50 ng/mL FGF7. 1 mL of medium per well and day with double concentration of reagents.
143. Perform a 50% of medium change as described before. Store the rest of the medium at 2°C–8°C until the day of intended use.
144. Incubate the plate at 37°C and 5% CO_2_ in an orbital shaker at 100 RPM inside the incubator for 24 h.
145. On day 2, change medium to pre-made and pre-warmed medium.
146. Incubate at 37°C and 5% CO_2_ in an orbital shaker at 100 RPM inside the incubator for 24 h.

#### *Stage 3:* Differentiation to immature beta cells

**Timing: 5 days**

147. Prepare the volume of Stage 3 medium required for day 1 and 2: DMEM 25 mM Glucose with 1× B27, 500 nM LDN-193189, 30 nM TBP, 1 μM ALKi II, 25 ng/mL FGF7. 1 mL of medium per well and day with double concentration of reagents.
148. Daily perform a 50% of medium change as described before. Store the rest of the medium at 2°C–8°C until the day of intended use.
149. Incubate the plate at 37°C and 5% CO_2_ in an orbital shaker at 100 RPM inside the incubator for 24 h.
150. In day 3 and 5, prepare fresh Stage 3 medium and continue performing daily 50% medium changes.

#### *Stage 4:* Differentiation to mature beta cells

**Timing: 7 days**

151. Prepare the volume of Stage 4 medium required for day 1–7, since this medium does not contain growth factor and can be stored for longer: DMEM 2.8 mM Glucose, 1× Glutamax, 1× non-essential amino acids. 1 mL of medium per well and day with double concentration of reagents.
152. Daily perform a 50% of medium change as described before. This medium can be stored at 2°C–8°C for two weeks.
153. Incubate the plate in an orbital shaker at 100 RPM inside the incubator for 24 h until day 7 of Stage 4.
154. Harvest spheroids for further analysis.
  a. Using a serological pipette or a 2 mL stripette, gently remove approximately the half of the medium from the well.
  b. Dispense the medium back directly to the surface of the well to dislodge the spheroids from the microwells. Do not pipette too much to avoid the deterioration of the aggregates.
  c. Collect the suspension in a conical tube.
  d. Dispense 1 mL of warm Stage 4 medium across the surface of the well to dislodge the possible remaining aggregates and collect it in the conical tube.
  e. Check the wells under the microscope to ensure all the aggregates have been collected. If there are some remaining, repeat the wash with medium.
  f. Optional: Aggregates can be counted at this point with a hemocytometer to determine the yield.

### Step 4B: Differentiation of pancreatic progenitors into beta cells in 3D organoids

**Timing: 16 days**

This section describes an alternative to the previous section for the differentiation of expanded pancreatic progenitors into beta cells, using domes of Matrigel where they have expanded to maintain the 3D structure. The timing and medium used for this section are the same as those reported in the previous one, adapted from (Russ et al., 2015; Trott et al., 2017), with the exception that it is not necessary to wait 24 h to begin the differentiation. This process presents advantages over the aggregates protocol: the medium change is much easier to perform since domes are bigger, they are easier to see and thus more difficult to aspirate or lose; the 3D structure is easier to maintain, since organoids are not disrupted during the process, and they are already formed when it begins; there is no need of special plates. However, the main disadvantage falls on the higher number of mesenchymal cells present in the domes, leading to a relative lower final percentage of endocrine cells.

***Note:*** 6-well plates are used for this protocol. Up to 10 domes of Matrigel may be grouped in the same well.

155. Prepare the volume of Stage 1 medium required for day 1 and 2, described in 156. 3 mL of medium per well and day.
156. Collect domes of Matrigel with expanded pancreatic progenitors in a 6-well plate.
  a. Aspirate the medium.
  b. Wash with D-PBS being careful not to disturb the domes.
  c. While in D-PBS and using the plunger of a 1 mL syringe, gently detach the Matrigel domes from the plate, paying attention not to break them.
  d. Cut approximately 1 cm of a 1 mL pipette tip with sterile scissors, making it large enough to fit the Matrigel domes without breaking them.
  e. Gently aspirate the Matrigel domes with the cut pipette tip and transfer them to a new 6-well plate. Domes that will undergo the same treatment may be grouped in the same well up to 10.
  f. Remove the D-PBS left in the new plate and add 3 mL of warm Stage 1 medium.
157. Incubate the plate at 37°C and 5% CO_2_ in an orbital shaker at 100 RPM inside the incubator for 24 h.
158. The medium changes are the same as in the previous section until day 7 of stage 4, with 24 h of incubation in an orbital shaker at 100 RPM between each change.
159. Harvest domes for further analysis.
  a. Matrigel domes can be directly washed with D-PBS and fixed with PFA for further imaging analysis.
  b. If cells retrieving is necessary for nucleic acid or protein extraction, treat the Matrigel domes with 250 μL of TrypLE per dome for 5 min at 37°C. Add 250 μL of D-PBS and pipette up and down to disaggregate the cells. Centrifuge for 5 min at 300 g at RT and aspirate the supernatant.

## Expected outcomes

During the differentiation process from hiPSCs to PP, the expression of the different and specific markers varies, being the most important way of validating the differentiation quality. At DE stage, OCT4 expression must be almost gone, being present in lower than 10% of cells. On the other hand, SOX17 should be expressed in more than 90% of the total population. When PF stage is reached, HNF1B should be present in a high percentage of cells and PDX1 begins to be expressed. At PP stage, PDX1 reaches a peak of expression with 70–80% of PDX1-positive cells and NKX6.1 is also expressed in a 30–50% of the cells (Figure 2). These two factors have been shown as indispensable for endocrine pancreas differentiation and failure in getting the proper percentage of positive cells, as shown in Figure 2E, would also suppose a failure in differentiation (Memon et al., 2018). For the endocrine differentiation, insulin is expected to be expressed in approximately 25% of all cells, while somatostatin and glucagon are also expressed but in a lower number of cells (Figure 4).

**Figure 3:**
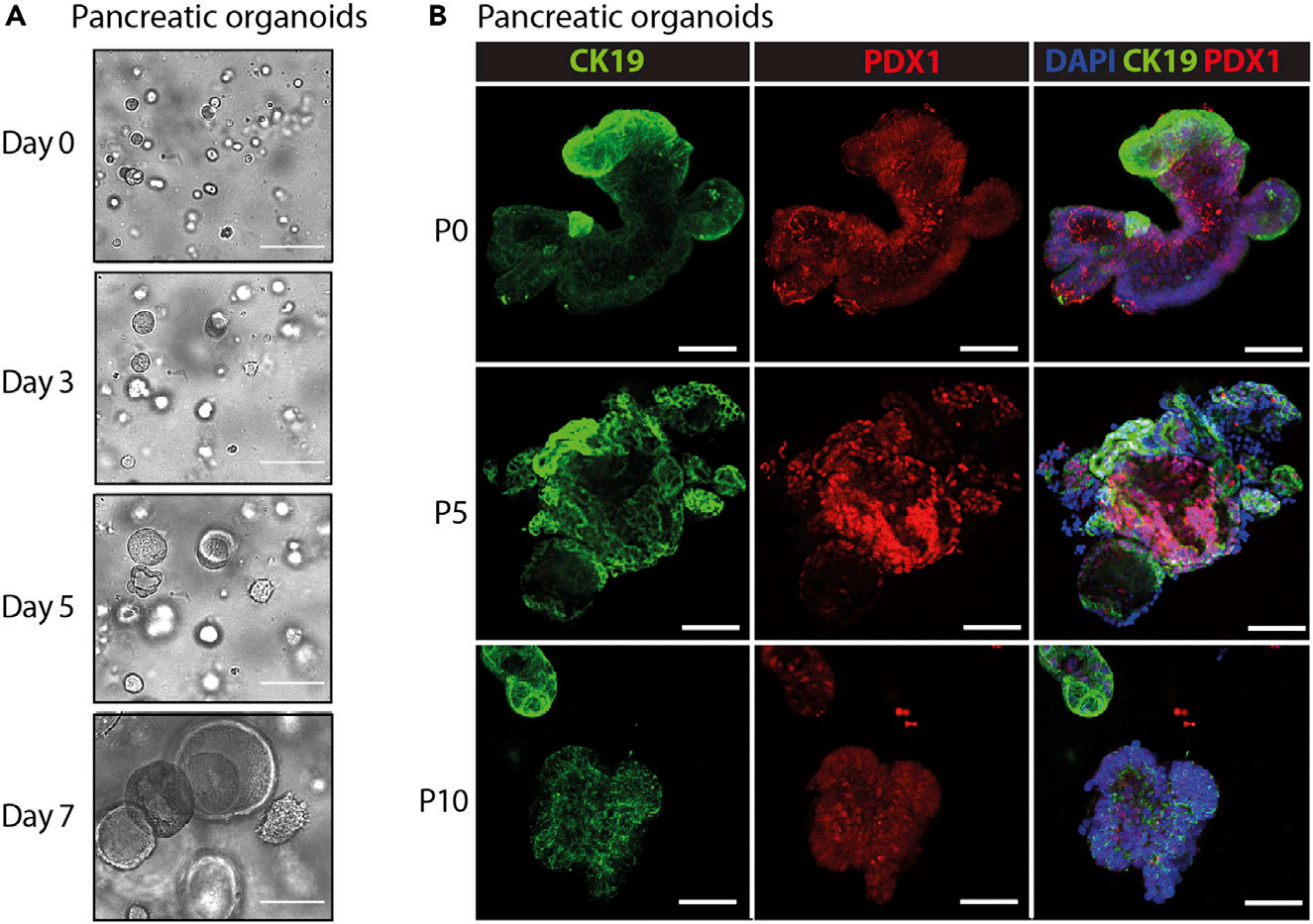
Characterization of pancreatic organoids (PO) (A) Brightfield images of pancreatic organoids growing at different time points after seeding. (B) Immunofluorescence characterization of pancreatic organoids showing PDX1 and CK19 expression at various differentiation stages. Scale bars, 100 μm (A) and 50 μm (B).

**Figure 4:**
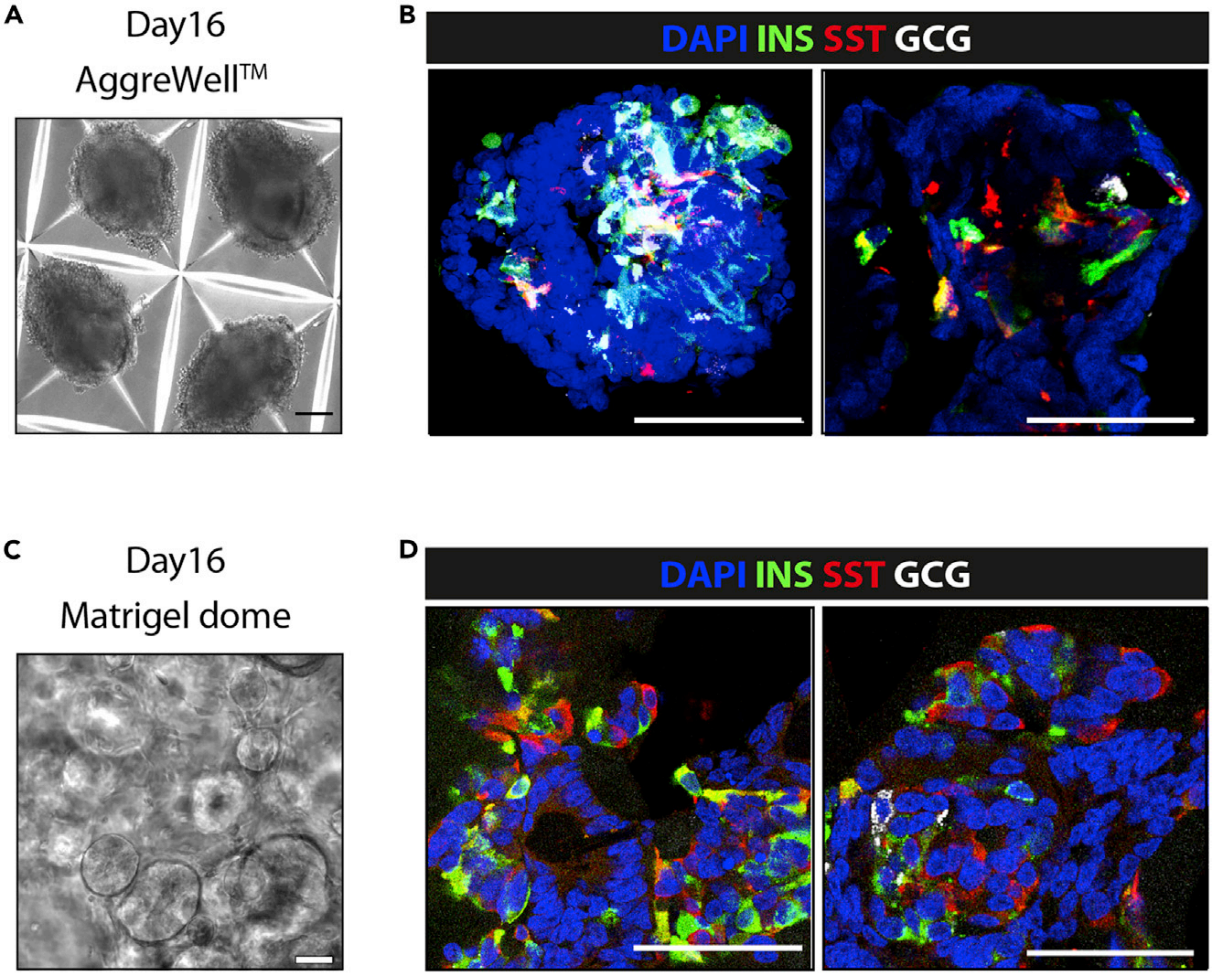
Characterization of endocrine cells at the final differentiation stage (A) Brightfield picture of aggregates after 16 days of differentiation. (B) Immunofluorescence for insulin, somatostatin and glucagon at the end of differentiation in aggregates. (C) Brightfield picture of a dome of Matrigel after 16 days of differentiation. (D) Immunofluorescence for insulin, somatostatin and glucagon at the end of differentiation using Matrigel. Scale bars, 100 μm (A and C) and 50 μm (B and D). Adapted from Cujba et al. (2022).

Regarding the expansion of organoids, small round organoids might be observed 2 days after their formation. These grow over 7–10 days showing bigger organoids with the typical round shape and lumen (Figure 3). A small percentage of mesenchymal cells (around 5%) not forming organoids is expected, but this must not be higher, or it would impair organoids growth and differentiation. High percentage of pancreatic progenitors in the culture and fresh medium every 2 days should be enough to keep mesenchymal cells in low proportion. If not, since they are single cells, size separation based on shorter TrypLE incubation and centrifugation, or selection based on E-Cadherin can be performed to deplete mesenchymal cells from the culture.

## Limitations

We have found that approximately 10% of the differentiation experiments did not achieve proper expression of the specific markers for pancreatic progenitors or beta cells, meaning a poor differentiation process. This may be a result of a high percentage of mesenchymal cells, due to bad conditions of the initial hiPSCs or the reagents used for the differentiation. Another limitation of this method is the extra care needed when aspirating and dispensing medium to proceed with the differentiation from pancreatic progenitors without losing too many cells or Matrigel domes in the process (troubleshooting 4 and 5). The formation of beta-like cells using suspension methods can result in loss of significant number of cells during medium change throughout the 17-day period.

## Troubleshooting

### Problem 1

Cell detaching during PP differentiation at the late stage, in the last few days of Stage 4. Seeding density have been too high at the beginning of the differentiation and the cells may detach as a full layer from the plate as a result (step 74).

### Potential solution

Optimize the seeding depending on the growth rate of the cells. Even if the cells detached, they can be used to form organoids as soon as the event is observed, to avoid complete loss of the differentiated cells. Observe if organoids grow in the next 7–10 days.

### Problem 2

Organoids expand at a very slow rate throughout the 7–10 days expansion phase. The cell density was too low (step 94).

### Potential solution

Dissociate the Matrigel domes with the existing organoids/cells and seed then in a smaller number of domes per well. Reduction by ¼ or ½ in the number of Matrigel domes per well would suffice to ensure optimized organoids expansion in the next differentiation stage. Use Y-27632 at 10 μM during the first two days after dissociation to improve their growth.

### Problem 3

Differentiation efficiency is too low at PP stage (step 83; PDX1 expression levels are not high enough or PDX1 is expressed only in few cells) or there is a high cell death rate and a very low number of cells at the end of the protocol.

### Potential solution

This problem may be due to an inappropriate cell confluency at the beginning of the differentiation, a suboptimal quality of the reagents or differentiation potential of the cells used. Cells should be seeded as 500,000 cells/well for 12-well plates and 42,000 cells/well for 96-well plates, but optimization of this ratio must be performed for each cell line. Use a fresh aliquot of all the necessary reagents for the differentiation process. Low passage (P0-P5) cells are recommended to improve differentiation efficiency.

### Problem 4

Differentiation efficiency is too low at beta cell stage (steps 154 and 159), hormones are not expressed.

### Potential solution

This may be due to an inappropriate cell confluency at the beginning of the differentiation, a suboptimal quality of the reagents or differentiation potential of the cells used. Domes of Matrigel must be confluent enough and organoids must be big enough before starting the differentiation. In the case of aggregates, they must have enough cells. To avoid this issue, leave the organoids grow for longer and seed a higher number of cells for the aggregates. Also, use a fresh aliquot of all the reagents necessary for the differentiation. Low passage hiPSCs are recommended to improve the differentiation efficiency.

### Problem 5

The number of aggregates at the end of differentiation (step 154) is too low.

### Potential solution

As explained in the limitations section, extra care is needed when aspirating and dispensing medium to proceed with the differentiation from the pancreatic progenitors without losing too many cells, especially when grown as aggregates. It is crucial to perform medium changes as careful as possible, check the cells under the microscope every time, and centrifuge the plate in case of doubt.

## Resource availability

### Lead contact

Further information and requests for resources and reagents should be directed to and will be fulfilled by the lead contact, Rocio Sancho (rocio.sancho@kcl.ac.uk).

### Materials availability

This study did not generate new unique reagents.

## Data Availability

This study did not generate/analyze [datasets/code].
